# Revolutionizing Health Care Analytics Through Data Democratization at America’s Largest Municipal Health Care Delivery System

**DOI:** 10.63116/ahisp-26-003

**Published:** 2026-05-28

**Authors:** Bharti Sharma, Adnan Abdul, Hnin Phyu, Maninder Sarao, Maryam Nunez

**Affiliations:** 1 Department of Data and AI, New York City Health + Hospitals

**Keywords:** Data Champions, Data Democratization, Data Academy/Literacy, Data Governance

## Abstract

In the rapidly evolving landscape of health care data utilization, achieving data democracy is crucial for enhancing organizational agility and decision-making effectiveness. In this article, the authors document the journey toward data democracy at America’s largest public hospital system, highlighting the strategic alignment among leadership and stakeholders to secure buy-in and commitment. Beginning with early adopters and an initial roadmap, the initiative progressed through iterative updates informed by evolving strategies and stakeholder input. A crucial element of this journey was the establishment of *data champions*. These champions were thoughtfully engaged and diligently trained by the data and analytics team through persistent efforts. In this article, the authors explore key areas of focus for data champions, including the establishment of a data academy, development of support mechanisms, formulation of governance policies and technical guardrails, curation of structured data assets, and facilitation of access to tools and technology. Challenges encountered in data integration, accessibility, and quality underscored the critical role of robust governance structures and policy frameworks in sustaining data-driven decision-making. The insights shared in this article aim to guide other organizations in fostering a data-informed culture, learning from both challenges and successes.

## Introduction

Health care today sits at the intersection of complexity and urgency. Every clinical, operational, or financial decision depends on data; yet, it remains siloed, delayed, and inaccessible. This challenge reflects a broader organizational phenomenon in which data access has historically been limited to specialized teams, creating bottlenecks that inhibit timely decision-making and operational performance.^1^ At America’s largest municipal health care system, this means weeks-long waits for reports and ad hoc facility solutions, as well as limited visibility to the leadership.

We knew that to truly serve our patients and communities, data could no longer be the privilege of a few; it had to become a shared language, accessible, and actionable by all. That belief became the foundation of our journey toward data democratization.

For the purposes of this article, we define *data democratization* as “the structured enablement of secure, governed, and technology-supported data access combined with ongoing data and technology literacy and operational support, so that users can independently generate reliable insights while maintaining compliance, quality, and organizational alignment.” This definition emphasizes that democratization extends beyond simple data access; it requires intentional governance frameworks, scalable technology administration, and sustained workforce development to ensure responsible and effective use.

In this article, the authors chronicle that journey—our vision, missteps, turning points, and triumphs. At its center are the data champions—frontline advocates trained and empowered to bring data into the daily fabric of decision-making. What began as a small experiment evolved into a system-wide movement, reshaping not only how we use data but also how we think about collaboration, equity, and innovation.

### Vision and Strategy: From Siloed Data to Shared Ownership

Our vision was simple yet bold: empower every decision-maker with data they could trust and use to drive better outcomes. This was not just about providing access—it was about making data actionable and integral to every decision, across all levels of the organization.

Our strategy rested on 3 foundational pillars:

- *Empowering individuals through data education*: We launched a data academy designed to build data literacy and confidence across the organization. By equipping employees at every level with the skills to understand and work with data, we ensured that data-driven decision-making became second nature.
- *Enabling collaboration through champions*: We embedded data champions within each site to act as both advocates and experts. These individuals bridged the gap between business users and technical teams, ensuring that data were used effectively and responsibly and aligned with organizational goals.
- *Fostering innovation through technology and governance*: To ensure that our democratization efforts were secure and sustainable, we built strong governance frameworks. These guardrails guaranteed that data remained high-quality, accessible, and compliant, allowing us to innovate without compromising on security or standards.

This multidimensional approach aligns with findings in the literature that successful data democratization in health care is sociotechnical in nature—requiring not just technology and access but also workforce capability development, clear governance structures, and operational support to translate data into actionable clinical and administrative insights.^2^

**Figure 1. Data champion program value P.**
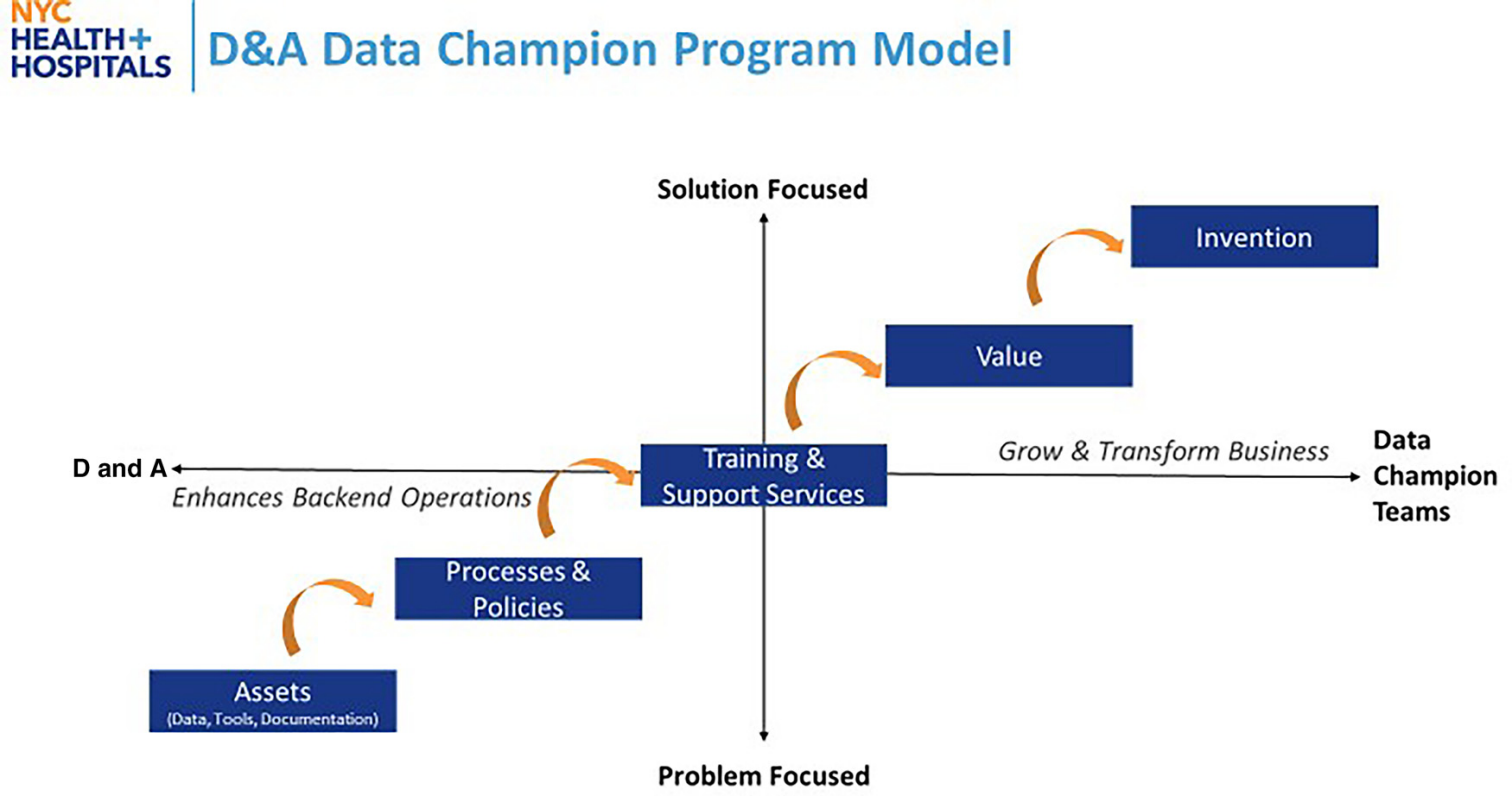


We leveraged the data champion value model to articulate clear benefits and address organizational gaps through a structured value proposition. As illustrated in Figure 1, the model outlines the data and analytics (D&A) team offering building blocks from foundational assets and governance to training, value realization, and innovation, thereby providing business leaders with a roadmap to drive both operational efficiency and transformation. This approach enabled CEOs and senior executives to become active partners, clearly signaling that the effort was not merely an IT initiative but also a broader cultural shift in how decisions are made, care is delivered, and public health outcomes are improved.

### Building the Minimal Viable Product (MVP): Birth of the Data Champion Program

In 2021, we found ourselves at a pivotal moment. Our health care facilities were experiencing a stark division in how they engaged with data. Some were ahead of the curve, forging their own path with customized reporting and analytics, but without a clear framework to guide them. Others were stalled, trapped in a holding pattern, waiting for help from the central D&A team. The long queues and resource bottlenecks became a growing pain that threatened to slow down progress across the system.

It was clear that the system was not working and change was necessary. So, we conducted a series of candid discussions with facility leaders, executives, and key stakeholders to understand the barriers they faced and to identify what was needed to move forward.

The responses were strikingly consistent, revealing the underlying challenges: unclear definitions of analytical maturity, heavy reliance on central data teams, and a need for data education in addition to tools.

These responses struck a chord. The problem was not merely access to tools or receiving support; it was about empowering the facilities to become self-sufficient. The real solution lay not in more resources but in fostering education, ownership, and the ability for local decision-making.

**Figure 2. Data champion program offerings.**
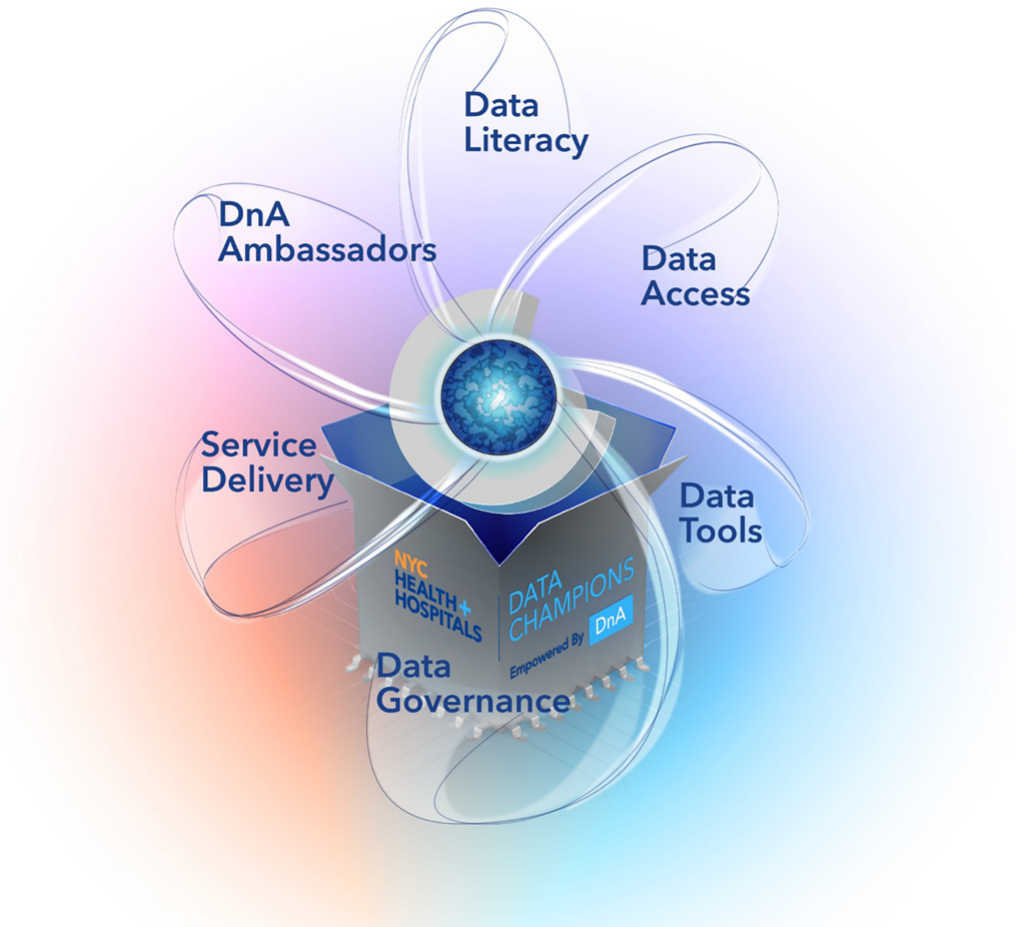


From these insights, the data champion program was established to return the power of data to those who needed it most: the people working at the local level. The program’s goal was to provide each facility with training and appoint its own data champions who were local experts familiar with the unique challenges and opportunities of their sites. These champions were empowered to make decisions and address issues locally, with ongoing support from the central D&A team. Figure 2 illustrates the core components of this model, highlighting how data literacy, access, tools, governance, service delivery, and data ambassadors (support) work together to enable local ownership and enterprise alignment.

The data champion program offers customized, instructor-led training through our data academy, which is designed to meet the unique needs of each facility. Participants not only gain knowledge but also demonstrate their proficiency through examinations that ensure mastery of key data skills.

In addition, the program provides access to powerful analytics tools such as Tableau, Snowflake, and EPIC, as well as curated data sets that reduce technical barriers and computational costs. To ensure consistency and compliance, we have also implemented standardized processes, policies, and governance frameworks for data management, ensuring that our data are secure and accessible.

However, the program extends beyond training and access, with ongoing support serving as a key element. Data champions receive continuous learning opportunities, consultations, and troubleshooting assistance to help them overcome challenges and innovate within their teams.

In this article, we dive into each of these offerings in more detail, exploring how the data champion program empowers teams, fosters innovation, and drives sustainable growth through data-driven decision-making.

### The Hub-and-Spoke Model: Strategy Meets Execution

**Figure 3. Enterprise data analytics and artificial intelligence hub-and-spoke model.**
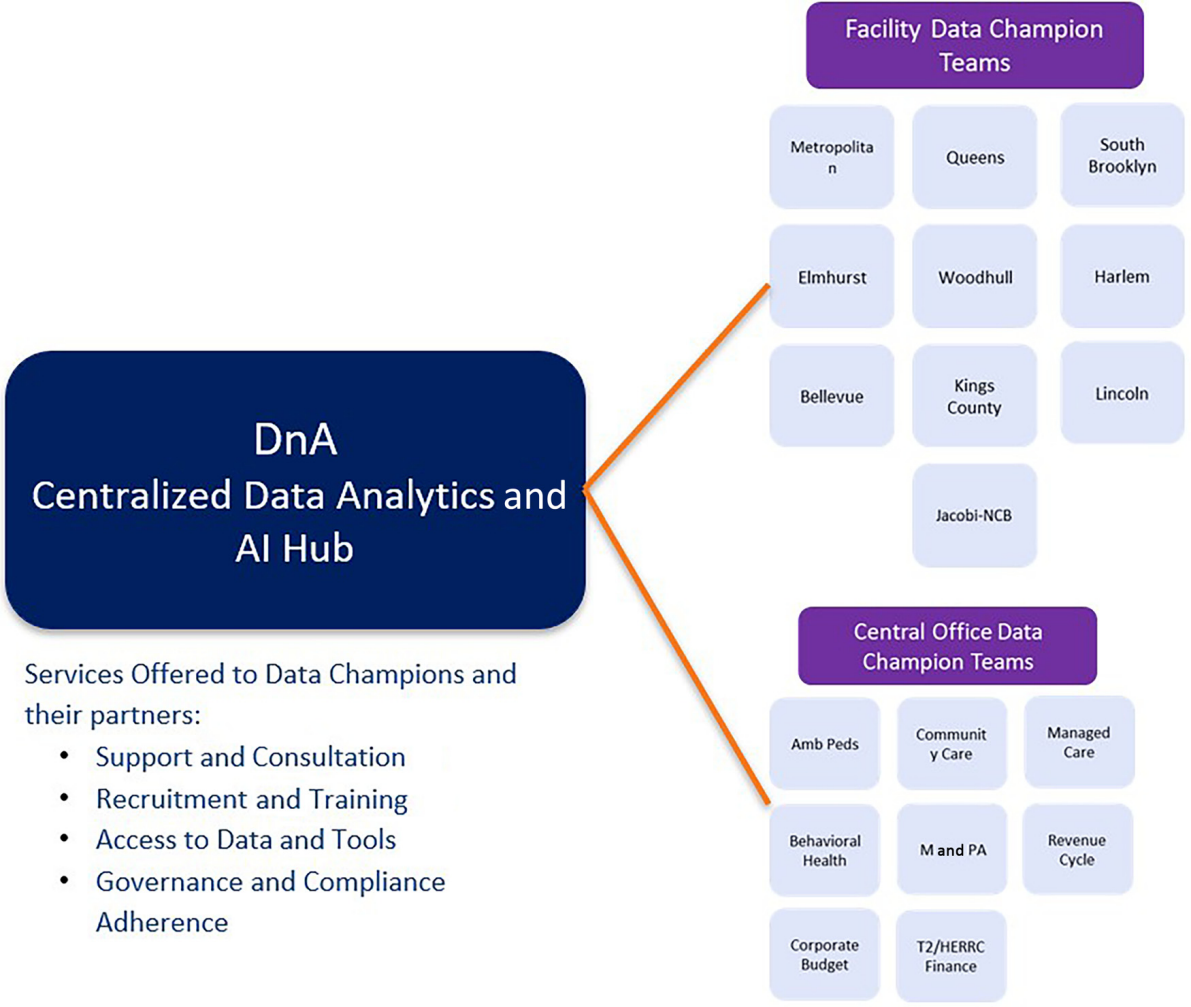


To make this vision a reality, we adopted a hub-and-spoke model that balanced the strategic needs of the enterprise with the day-to-day realities of each facility. The central D&A office would serve as the hub—responsible for setting overarching strategies, maintaining governance standards, and developing enterprise-wide tools such as dashboards and reporting frameworks, as illustrated in Figure 3.

The spokes were the local data champions at each facility, acting as the boots on the ground. They would take the strategic vision and translate it into local action: developing facility-specific dashboards, providing operational insights, and making real-time, data-driven decisions that aligned with the broader organizational strategy.

This model created a seamless synergy between the central and local teams. The central office provided consistency, governance, and high-level direction, ensuring that all facilities were working with standardized, high-quality data. Meanwhile, data champions operated quickly and nimbly within their own facilities, driving change at the local level.

## Discussion: Data Academy

### Standing Up the Data Academy: Empowering Champions Through Education

At the core of our data transformation journey was training—the essential catalyst that equips our teams to harness the full power of data and drive actionable insights across the organization. The data academy became the driving force behind this effort, providing structured pathways for data proficiency and supporting the development of data champions at each facility.

The data academy was established to offer foundational training in EPIC and Tableau, which are the key platforms used by our teams for reporting and informed decision-making.

### A Tailored, Multimodal Learning Experience

We intentionally designed the curriculum to be accessible and engaging, thereby offering multimodal learning formats that could support different learning styles. Through a mix of self-paced videos, instructor-led virtual sessions, workbooks, and office-hour consultations, we ensured that data champions could build their skills progressively, with hands-on practice reinforcing their learning.

The initial courses covered:

- *Fundamental EPIC reporting*: To train teams on EPIC reporting capabilities.
- *Tableau basics*: To equip teams with the skills to visualize and analyze data in an accessible, interactive way.

These courses were rolled out in a cohort-based model, with new groups starting each quarter to promote peer learning and foster a sense of community. Upon completing the course and passing the proficiency examinations, participants were granted the title of “NYC Health + Hospitals Proficient,” reflecting not only their newly acquired skills but also their commitment to data-informed decision-making.

### Identifying Gaps and Evolving Training

As we continued to roll out the program, we quickly realized that the initial training offerings although valuable were not enough to address the growing complexities of our data needs. Although EPIC and Tableau provided essential tools, many data champions expressed a need to go beyond basic usage. They needed more flexibility and depth in their data handling capabilities.

Based on this feedback, we introduced Advanced Tableau to deepen visual analytics skills and SQL training to help our champions to not only access data but also query and manipulate data to solve complex problems.

### The Evolution: Advanced Tableau and SQL

In response to the evolving needs of our teams, we expanded the curriculum. Advanced Tableau was added to help data champions create more sophisticated visualizations, and SQL Fundamentals was introduced to enable them to write queries and extract data. These courses equipped our champions with the technical expertise to progress from basic data consumption to data engineering, enabling them to optimize queries and create customized views tailored to their facility’s needs.

**Figure 4. Data champion skills and capability maturity scale.**
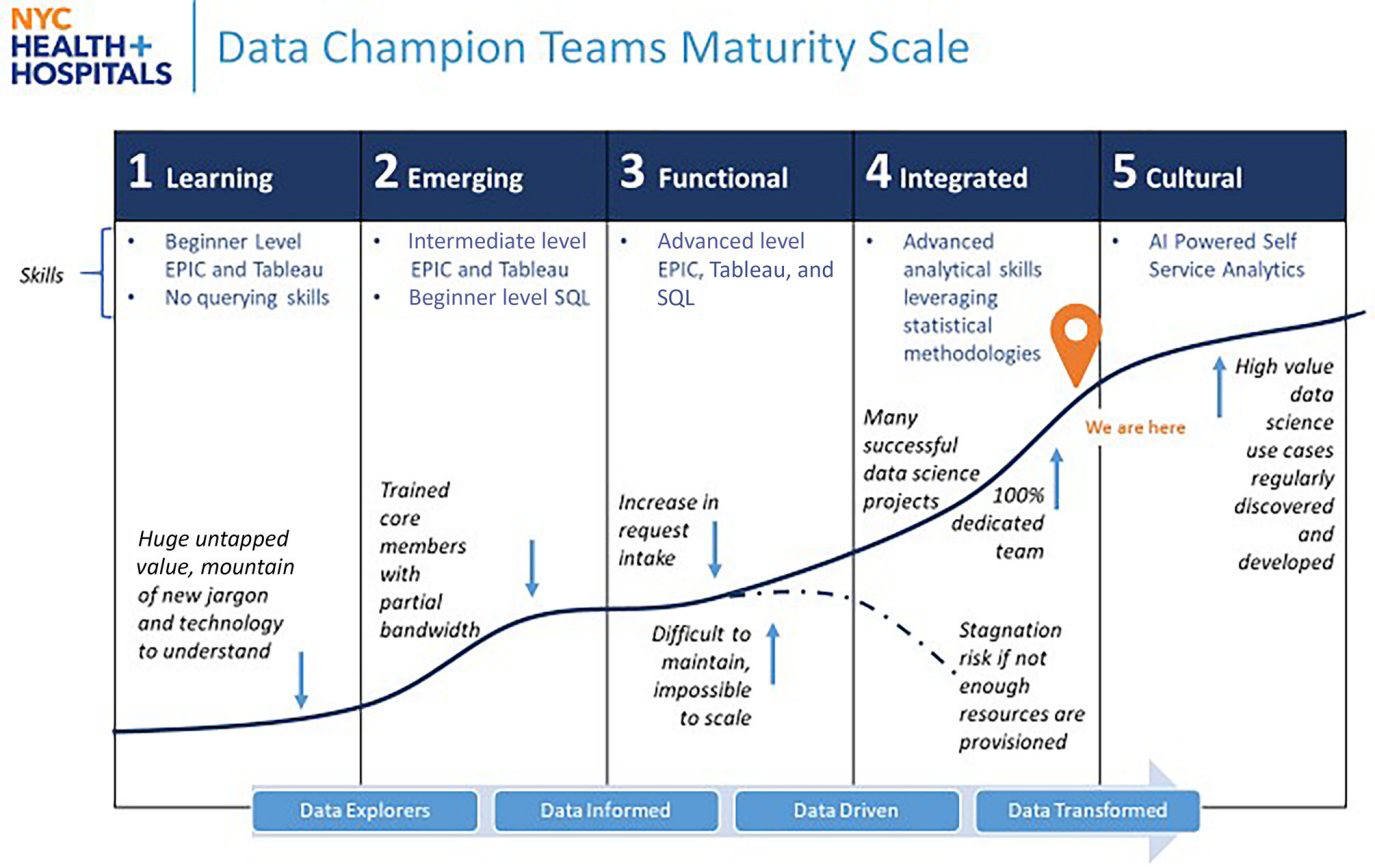


Figure 4 illustrates the data champion analytical maturity journey, showing the progression from zero prior analytical knowledge to an integrated state where data champions independently use EPIC, Tableau, and SQL to deliver scalable, high-value insights. At the *learning stage*, participants navigate EPIC reports and use basic Tableau visualizations capabilities without querying. In the *emerging stage*, they write simple SQL queries and modify dashboards. At the *functional stage*, they build dashboards from curated data sources and manage query joins independently. By the *integrated stage*, they apply advanced SQL, statistical methods, and performance optimization to develop scalable solutions aligned with enterprise standards. This progression reflects not only technical growth but also increasing ownership and analytical confidence.

### The Impact: Scaling Data Literacy Across the Organization

With each cohort of data academy graduates, we were scaling data literacy across the organization. Graduates earned the NYC Health + Hospitals Proficient title, which granted them access to the necessary data tools and resources. This was about not just having technical skills but also creating a culture of data where every decision was supported by accurate, timely information.

### A Model for Continuous Data Empowerment

The data academy became more than just a training program; it became the foundation of a sustainable, self-sufficient data culture across the organization. Through it, we have been able to upskill our workforce, foster peer-driven learning, and ensure that every data champion has the tools they need to contribute to the organization’s data-driven mission.

## Fostering Innovation Through Data, Technology, and Governance

### Data Architecture: Laying the Data Foundation

When NYC Health + Hospitals (NYC H + H) launched its data democratization program, the goal was clear—empower decision-makers with trusted, timely data. However, as access expanded, we quickly learned that empowerment needed structure. In health care, where accuracy, privacy, and security are paramount, the foundation had to be strong.

The journey began with 3 data champion teams in Wave 1, using 15 key data assets made available in Snowflake. These data assets were structured, curated, centrally governed and ratified, analytically derived from the enterprise data warehouse (EDW) and designed for secure, scalable analysis. They encompassed multiple domains, including clinical data (e.g., patient encounters, diagnoses, and laboratory results), operational data (e.g., staffing, scheduling, and throughput metrics), and administrative data (e.g., finance, billing, and resource utilization). This initial wave tested how securely and efficiently data could be shared at scale. After collecting feedback through user surveys, we refined our approach—strengthening data policies and streamlining access controls.

**Figure 5. Snowflake data controls architecture. AD, Active Directory; EDW, enterprise data warehouse.**
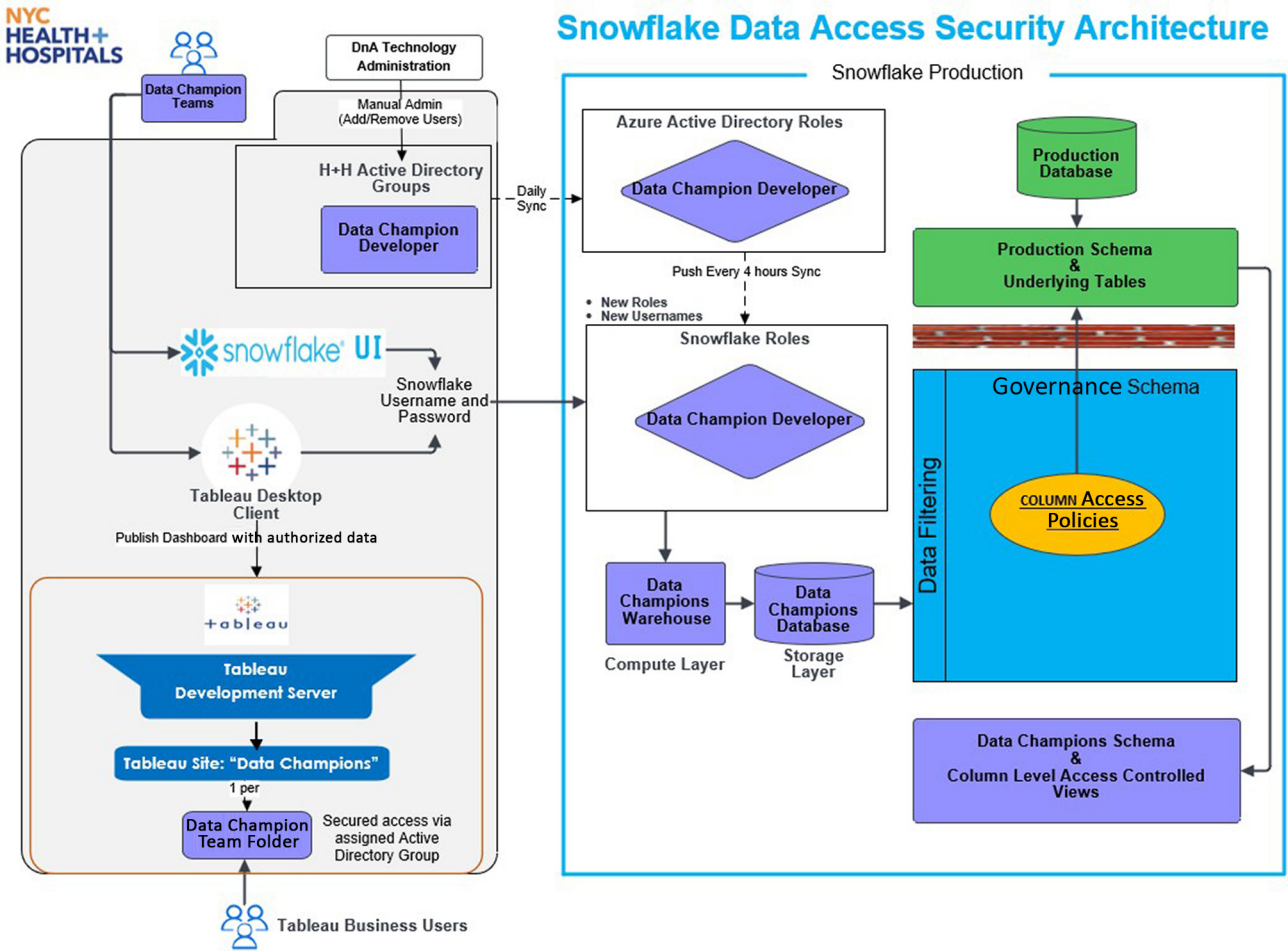


Subsequent waves expanded the program to include all EDW/analytics layer tables through a dedicated data champion schema in Snowflake, built as views directly referencing the EDW layer to ensure real-time consistency while embedding behavioral health and Substance Use Disorder (SUD) policies for full regulatory compliance. As shown in Figure 5, governance is embedded directly into the architecture: Production tables remain protected within the EDW schema, and the EDW_BI schema delivers curated, column-level access-controlled views for self-service analytics. Sensitive domains are secured through policy-based data filtering, maintaining compliance without compromising analytical flexibility.

### Data and Tools Access: Technology Guardrails for Smarter Use

To make data truly usable, we combined accessibility with control. Each data champion team was assigned a dedicated Tableau site within the sandbox environment, allowing them to securely publish dashboards. Access to each site was restricted to the respective facility’s users through active directory (AD) groups, while teams had full autonomy to manage their own users and content.

In Snowflake, each team was provisioned with a dedicated virtual warehouse, carefully sized for performance and cost optimization. Guardrails were applied to keep usage efficient—queries were limited to 20 minutes of execution time, and auto suspension was triggered after 10 minutes of inactivity to control compute costs.

To maintain alignment and simplify oversight, the same AD groups were used for both Snowflake and Tableau access, allowing unified monitoring, consistent permissions, and streamlined user management.

### Governance as an Enabler

Governance was never about restrictions but fostering confidence. As shown in Figure 5, we established role-based access controls to ensure that users only accessed the data necessary for their role. AD groups were synchronized with Snowflake roles, creating a structured, auditable access model. Each participant signed a data usage agreement, and audit logs tracked all activity for full transparency and accountability. This governance framework became the enabler for responsible innovation. Standardized data structures, clear policies, and aligned access models empowered teams to collaborate freely while maintaining compliance and trust. Silos began to dissolve, enabling cross-functional teams to share insights effortlessly.

In addition, a centralized data governance committee was formed, consisting of data stewards and executive data domain leaders, to verify and standardize metrics, measures, and key performance indicators, (MMKs) for future use by NYC H + H citizens.

As per Figure 5, this architecture brings together centralized governance, role-based access, and dedicated, cost-controlled compute to deliver secure, facility-level self-service analytics with built-in regulatory protections across the enterprise.

## Results

With more than *250 data champions* embedded across various departments, these empowered individuals have created over *1,000 reports* and *300 Tableau dashboards*, significantly reducing the resource burden on our central D&A office.

### Quantifiable Impact

Before the data champion program, the central office D&A team was responsible for producing all dashboards and reports for the entire system, managing a backlog of over 300 outstanding requests.

By enabling data champions to independently create reports and dashboards, we have achieved substantial *cost and resource savings*. Historically, creating a specialized dashboard would take *3 to 4 weeks* (roughly *120-160 hours*) and cost about *$7500 to $10,000* per dashboard when leveraging specialized analysts. With *300 dashboards* created by data champions, this results in *$2.25 million to $3 million* cost savings.

Reports typically take about *1 week* (approximately *40 hours*) to build, with an analyst’s time costing roughly *$2500* per report. Given that data champions generated *1000 reports*, this results in savings of additional *$2.5 million*.

*Total estimated savings* from dashboard and report creation alone: *$4.75 million to $5.5 million* annually.

### Broader Organizational Impact

The data champion program has fundamentally shifted how we approach data. With more than 400 employees now data-trained and proficient, these individuals are actively using data to drive meaningful impact in their respective areas:

- *Improved operations*: Data champions are streamlining processes and optimizing workflows within their teams, contributing to faster decision-making and increased efficiency.
- *Enhanced patient care*: By leveraging data, champions in health care-related departments have improved patient outcomes through more timely interventions and better resource allocation.
- *Quality and performance improvement*: Data champions are identifying performance gaps, helping us implement quality improvement initiatives that elevate both customer satisfaction and internal performance metrics.
- *Revenue generation and cost savings*: Data-driven insights are directly informing revenue-enhancing strategies, while also pinpointing areas for operational cost reductions and efficiency gains.
- *Risk reduction*: Data champions are proactively identifying potential risks, allowing us to mitigate challenges before they escalate, particularly in compliance, security, and operational domains.
- *Proactive problem-solving*: Shifting from a reactive to a proactive mindset, data champions are now anticipating challenges and driving improvements before issues arise, making our teams more agile and prepared.

### Strategic Impact on Central Team

The central D&A team has experienced a significant shift in focus, now able to dedicate their time to enterprise-level strategic initiatives that drive innovation and long-term business growth. Instead of being caught up in the daily demands of reporting, the central team is working on high-impact projects that push our data capabilities forward, enabling us to stay ahead of industry trends and continue to innovate.

## Conclusions

### Lessons Learned

Several key insights emerged from our data democratization journey, offering guidance for future initiatives and continued improvement efforts.

#### Investing in Human Capital: Beyond Platforms

Making data accessible requires more than just technology; it hinges on users’ ability to interpret and apply data effectively. Data democratization aims to empower all individuals within the organization, not just experts, to engage meaningfully with data. However, without adequate training and support, access can lead to underuse, misinterpretation, and resistance. Wang et al^2^ reported that data literacy is crucial for successful democratization. Tailored training programs at different proficiency levels are key to building user confidence and competence. When data literacy is prioritized, tools are used effectively and responsibly, making training an essential part of any data democratization strategy.

#### Balancing Governance and Agility in Data Democratization

Effective data democratization requires a governance model that safeguards data quality, privacy, security, and ethical use, while providing sufficient flexibility to support daily operations, audits, and quality improvement. From our experience, overly strict governance, such as centralized decision-making and rigid controls, can hinder adoption, reduce engagement, and limit innovation. Conversely, too little oversight can lead to data misuse, inconsistency, and regulatory noncompliance.

Research highlights the need for a balanced approach. Wang et al^3^ emphasized that successful data democratization depends on governance frameworks that balance trust, policy constraints, and the diverse needs of users.

#### Equity as a Foundation for Data Democratization

A key lesson from our health care data democratization efforts is that equity must be built into the strategy from the beginning, not addressed after implementation. Simply increasing access to data is insufficient; without a focus on equity, underserved groups may remain excluded.

We operationalized equity through a framework-guided approach:

- *Equitable access*: Ensuring all facilities and staff had access to standardized data tools, training through the data academy, and the appropriate permissions to use data safely and effectively.
- *Equitable participation*: Deliberately representing smaller or under resourced sites in data champion roles to ensure diverse perspectives and engagement across the system.
- *Equitable impact*: Monitoring measurable outcomes, such as the number of dashboards, reports, and initiatives generated across facilities, to confirm that benefits were distributed fairly and not concentrated in larger or better-resourced sites.

Without this framework, well-intentioned democratization efforts may inadvertently reinforce existing inequalities.^4^

#### Managing the Unexpected Through Iterative Implementation

Another critical lesson from our data democratization efforts was learning to expect the unexpected. Despite detailed planning and clearly defined objectives, we faced several unexpected challenges, such as data quality issues, regulatory constraints, end-user’s resistance, and system integration complexities. Rather than interpreting these disruptions as setbacks, we adopted an iterative, feedback-driven approach. This allowed for continuous adjustment and refinement of both technical infrastructure and support workflows based on regular feedback.

This approach relied on ongoing engagement with stakeholders, phased rollouts, and clear communication, which helped mitigate risk and foster trust among users.

These findings align with those of Alvarez-Romero et al,^5^ who showed that successful data initiatives rely on adaptive governance frameworks capable of evolving in response to new privacy regulations, shifting priorities, and technical constraints.

### The Road Ahead

As health care continues to advance, *data champions are expected to evolve into artificial intelligence (AI) champions*, playing a role in guiding the responsible use of AI, predictive models, and advanced analytics across clinical and operational settings. This shift involves not only using data effectively but also understanding how predictive models and advanced analytics are built, validated, and applied to support clinical decision-making, resource allocation, and population health management. AI champions will help ensure that these tools are used ethically, transparently, and in ways that align with patient-centered care.

To prepare for this expanded role, organizations will need to provide targeted training that builds both data and AI literacy. Staff should be equipped not just to access and visualize information but also to interpret predictive outputs and apply AI tools with confidence and care. Looking ahead, these AI champions will help bridge the gap between technical experts and frontline users, making sure technology serves people, not the other way around. Their leadership will be key in shaping a health care system where data, predictive analytics, and AI work in harmony to improve outcomes, efficiency, and equity.

### A New Era of Data-Driven Transformation at NYC H + H

As we reflect on the journey of building, scaling, and refining the data champion program, it is clear that what began as an ambitious vision has blossomed into a transformative force within NYC H + H. From the initial pilot projects to the full system-wide expansion, our commitment to empowering local teams with data, tools, and trainings has reshaped not only the way we make decisions but also how we view the role of data in health care.

This journey was about not just implementing technology but also empowering people—giving them the knowledge and tools to use data to drive change. The program’s success can be attributed to the collaborative efforts of our data champions, facility leaders, and the D&A team, each contributing to a shared goal of data democratization.

The scalability of this program means it is not just sustainable but also future-proof. We have built the infrastructure to continue evolving whether through the integration of advanced technologies, the ongoing development of training pathways, or our work with human resources to build career growth models. NYC H + H is now poised to thrive in an increasingly data-driven health care landscape.

Looking forward, our focus will be on refining our approach, expanding the reach of our champions, and creating even more opportunities for growth. We are committed to adapting and iterating, recognizing that the journey toward data democratization is never truly over.

## Funding

The authors received no external funding for this research.

## Disclosures

The authors declare no conflicts of interest.

## Author Contributions

Bharti Sharma confirms that all listed co-authors have made significant contributions to the manuscript and have reviewed and approved the final version.

CE Quiz
